# ACLY (ATP Citrate Lyase) Mediates Radioresistance in Head and Neck Squamous Cell Carcinomas and is a Novel Predictive Radiotherapy Biomarker

**DOI:** 10.3390/cancers11121971

**Published:** 2019-12-07

**Authors:** Eva-Leonne Göttgens, Corina NAM van den Heuvel, Monique C de Jong, Johannes HAM Kaanders, William PJ Leenders, Marleen Ansems, Johan Bussink, Paul N Span

**Affiliations:** 1 Radiotherapy and OncoImmunology laboratory, Department of Radiation Oncology, Radboud university medical center, 6525 GA Nijmegen, The Netherlandsmarleen.ansems@radboudumc.nl (M.A.); jan.bussink@radboudumc.nl (J.B.);; 2 Department of Biochemistry, Radboud Institute for Molecular Life Sciences, University Medical Centre, 6525 GA Nijmegen, The Netherlands; 3 Department of Radiation Oncology, The Netherlands Cancer Institute, Plesmanlaan 121, 1006 CX Amsterdam, The Netherlands; m.d.jong@nki.nl

**Keywords:** HNSCC, radiosensitivity, ACLY, predictive markers, DNA repair

## Abstract

Radiotherapy is an important treatment modality of head and neck squamous cell carcinomas (HNSCC). Multiple links have been described between the metabolic activity of tumors and their clinical outcome. Here we test the hypothesis that metabolic features determine radiosensitivity, explaining the relationship between metabolism and clinical outcome. Radiosensitivity of 14 human HNSCC cell lines was determined using colony forming assays and the expression profile of approximately 200 metabolic and cancer-related genes was generated using targeted RNA sequencing by single molecule molecular inversion probes. Results: Correlation between radiosensitivity data and expression profiles yielded 18 genes associated with radiosensitivity or radioresistance, of which adenosine triphosphate (ATP) citrate lyase (*ACLY*) was of particular interest. Pharmacological inhibition of ACLY caused an impairment of DNA damage repair, specifically homologous recombination, and lead to radiosensitization in HNSCC cell lines. Examination of a The Cancer Genome Atlas (TCGA) cohort of HNSCC patients revealed that high expression of *ACLY* was predictive for radiotherapy failure, as it was only associated with poor overall survival in patients who received radiotherapy (hazard ratio of 2.00, 95% CI: 1.12–3.55; *p* = 0.0184). These data were further validated in an independent cohort of HNSCC patients treated with chemoradiation. Furthermore, patients with poor locoregional control after radiotherapy have significantly higher nuclear ACLY protein levels. Together, we here show that ACLY affects DNA damage repair, and is a predictive factor for radiotherapy outcome in HNSCC.

## 1. Introduction

Head and neck squamous cell carcinomas (HNSCC) are malignancies of the epithelium lining the nasal and oral cavity as well as the pharynx, and annually affects 650,000 people worldwide [1]. Together with surgery and (concurrent) chemotherapy, radiotherapy (RT) is an essential part of standard treatment for patients with HNSCC. Although RT is generally highly effective, tumors recur in a substantial number of patients with advanced stage disease [2]. In HNSCC originating in the oropharynx, the odds of treatment success are significantly affected by the human papillomavirus (HPV) status of the tumor. HPV-positive tumors constitute approximately 5%–60% of HNSCC cases, depending on the specific anatomical location [3]. HPV positive oropharynx tumors have been shown to be significantly more radiosensitive, and this is reflected in the patient population where HPV-positive patients have a strikingly better prognosis, locoregional control, and five year survival [4,5,6,7,8]. The mechanisms leading to radioresistance especially in HPV-negative patients have been extensively studied and have been attributed to DNA damage repair, cell cycle arrest, activation of oncogenes, microenvironmental changes, as well as changes in tumor metabolism [9].

Malignant cells/tissues have been shown to have a significantly different metabolism than non-malignant tissues and this is considered to be one of the hallmarks of cancers [10]. In addition, HNSCCs undergo extensive metabolic reprogramming upon malignant transformation, including others changes in oxidative phosphorylation, the tricarboxylic acid cycle, fatty acid metabolism, and increase of glycolysis [11,12,13]. These reports indicate that, overall, in cancer cells the utilization of glucose and glutamine as a primary energy source is enhanced, matching the leading theory that cancer cells switch to aerobic glycolysis to generate sufficient macronutrients to allow for cellular growth and proliferation [11].

Interestingly, metabolic reprogramming has previously been linked to changes in radiosensitivity, and has been shown to contribute to the development of radioresistant tumor cells. Increased aerobic glycolysis has been demonstrated to lead to elevated levels of lactate, pyruvate, and acetate, as well as changes in the redox state of cells. Overall, these changes affect the antioxidizing capacity of cells, enabling them to effectively scavenge DNA-damaging reactive oxygen species produced by ionising radiation [14].

In this study, we investigate the association between radiosensitivity and the metabolic gene profile in HNSCC. Hereto, we use a panel of HNSCC cell lines to identify clinically relevant metabolic transcripts that could confer radioresistance, irrespective of HPV status. We show that expression of ATP citrate lyase (*ACLY*), the enzyme converting citrate to oxaloacetate and acetyl-CoA, correlates with radioresistance in cell lines. Inhibition of ACLY led to impaired homologous recombination and increased radiosensitivity, which is in line with a recently described role for ACLY in the DNA damage repair pathway [15]. Furthermore, we show in two independent patient cohorts that patients who receive RT have a poorer prognosis in the case of high *ACLY* expression, and that nuclear ACLY may represent a novel target for radiosensitization in HNSCC.

## 2. Results

### 2.1. Multiple Metabolic Genes Associate with Radioresistance and –Sensitivity

**Figure 1:**
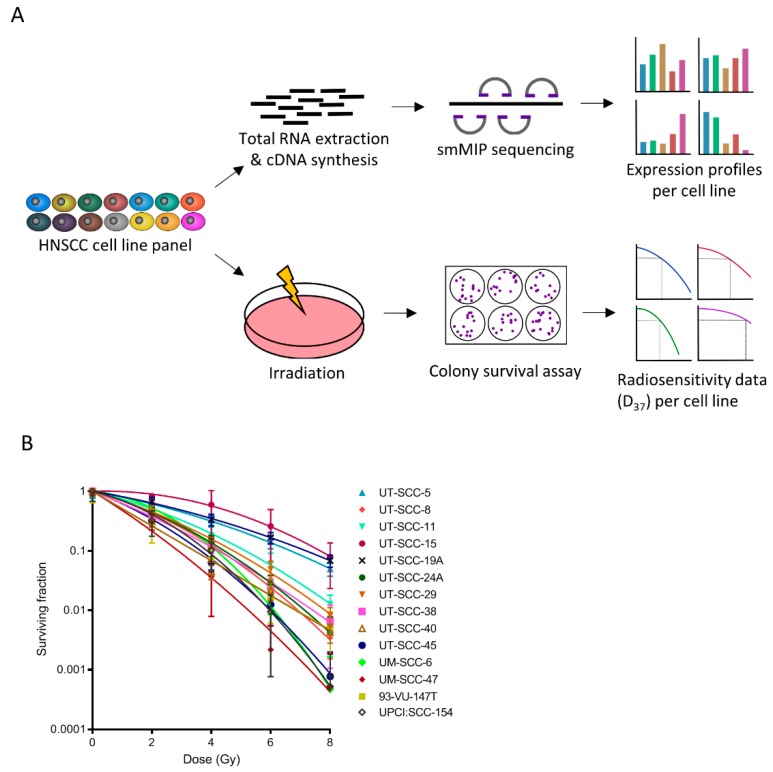
Characterization of 14 head and neck squamous cell carcinomas (HNSCC) cell lines. (**A**) Schematic representation of the approach to determine radioresistance or -sensitivity associated metabolic transcripts; (**B**) 10 HPV-ve (UT-SCC-5, 8, 11, 15, 19A, 24A, 29, 38, 40, and UM-SCC-6) and four HPV+ve (UT-SCC-45, UM-SCC-47, 93-VU-147T, and UPCI: SCC154) cell lines were exposed to 0, 2, 4, 6, or 8 Gy. Survival was determined by colony formation assay.

To investigate the link between radiosensitivity and the metabolic gene profile, we first characterized the radiosensitivity of a panel of HNSCC cell lines. This panel of 14 different HNSCC cell lines showed that there is a high variability in radiosensitivity between the lines (Figure 1A,B). Using a targeted sequencing approach by means of single molecule molecular inversion probes (smMIPs) for high-risk HPV types (HPV16, HPV18, HPV33, and HPV52) we validated the previously established HPV status in the cell lines (Supplementary Figure S1A). Using linear quadratic model fitting, the α and β, of each cell line were determined, which are the main parameters of intrinsic cellular radiosensitivity. Subsequently, the radiation dose permitting 37% survival (D37) was interpolated from the linear quadratic model (Supplementary Figure S1B). As expected, the D37 was significantly lower for HPV-positive HNSCC cell lines compared to HPV-negative HNSCC cell lines (*p* = 0.013) (Figure S1C), indicating that the HPV-positive cells were more sensitive to irradiation, confirming previous reports [4,8]. We then performed smMIP sequencing for approximately 200 metabolic and cancer-related targets [16,17] to investigate whether gene transcripts could be identified that correlate with radiosensitivity or radioresistance. When we compared the four most radioresistant and radiosensitive cell lines (UT-SCC-5, UT-SCC-15, UT-SCC-19A, UT-SCC-11 vs. UT-SCC-40, 93-VU-147T, UM-SCC-47 and UT-SCC-45), we found that in the former expression levels of the tricarboxylic acid cycle, glutamine metabolism, and lipogenesis pathways were upregulated (Supplementary Figure S1D). Using a Spearman’s rank test to test for significant correlation between the D37 and gene transcripts, 18 targets were detected that significantly correlated with either radioresistance or radiosensitivity (Table 1). Of these transcripts, 16 correlated positively with radioresistance, and two with radiosensitivity.

### 2.2. Inhibition of ACLY Affects DNA Damage Repair and Radiosensitizes HNSCC Cells

**Figure 2:**
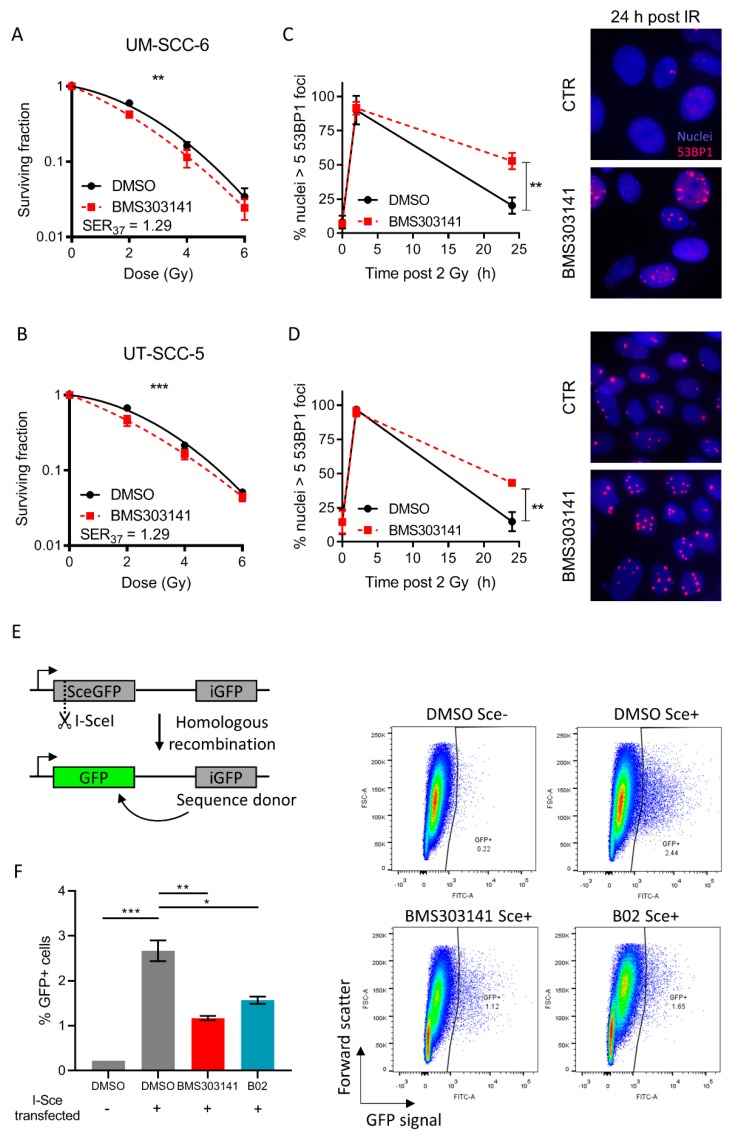
Inhibition of ACLY exacerbates radiation induced DNA damage in HNSCC cells and impairs homologous recombination. (**A**,**B**) UM-SCC-6 (A) and UT-SCC-5 (B) cells were treated with 5 µM BMS303141 for 6 h and irradiated with 2, 4, or 6 Gy. Survival was determined by colony formation assay. (**C**,**D**) Quantification of cells positive for 53BP1 foci (>5 53BP1 foci/nucleus in a single plane of view) in UM-SCC-6 (C) and UT-SCC-5 (**D**) cells. Shown are mean percentages of three biological replicates. Immunofluorescent representative images of examples of UM-SCC-6 or UT-SCC-5 cells, 24 h post irradiation, with or without 5 µM BMS303141 pre-treatment. Nuclei in blue, 53BP1 foci in red. (**E**) Homologous recombination assay. U2OS cells have been stably transfected with a construct containing a GFP gene containing a I-SceI restriction site (SceGFP) as well as a stop codon at the same site. Upon transfection with an I-SceI encoded plasmid, a double strand break is induced at the I-SceI site. Repair of this break via homologous recombination occurs through utilization of the internal GFP (iGFP) fragment downstream of the SceGFP. Successful homologous recombination results in the restoration of a functional GFP gene. (**F**) U2OS DRGFP cells were treated for 6 h with 5 µM BMS303141 or 5 µM BO2 (Rad51 inhibitor), and transfected with pCBASceI plasmid. 48 h post transfection, cells were harvested and the GFP positive fraction was determined using flow cytometry. Representative images from flow cytometry experiments are shown on the right, with DMSO Sce- as a negative control, and 5 µM B02 Sce+ as a positive control. In the graph, data are shown from two biological replicates.

As the ATP citrate lyase (ACLY) enzyme has recently been implicated to play a role in the DNA damage repair pathway [15] and it is one of our top hits to correlate with radiosensitivity, we hypothesized that ACLY may be relevant for further study. There was no significant difference in *ACLY* expression levels between HPV-positive and HPV-negative HNSCC cells, suggesting that HPV status is not a confounding factor (*p* = 0.09) (Supplementary Figure S2). To investigate whether ACLY indeed affects DNA damage repair and subsequent radiosensitization of HNSCC cell lines, we depleted *ACLY* expression via siRNA in the most radioresistant cell line, UT-SCC-15. siRNA mediated depletion of *ACLY* was confirmed 72 h after transfection (Figure S3A) and radiosensitised UT-SCC-15 cells to irradiation (Figure S3B). To move towards a more clinically realistic setting, we opted to further validate this using pharmacological inhibition of ACLY using BMS303141. UM-SCC-6 and UT-SCC-5 cells were treated with 5 µM BMS303141 for 6 h before irradiation, and thereafter colony survival was assessed as measure for radiosensitization. DNA damage repair was assessed by staining for 53BP1, an important repair factor which is rapidly recruited to sites of double-strand breaks [18]. BMS303141 treatment had a significant radiosensitizing effect on both UT-SCC-5 and UM-SCC-6 cells (sensitizer enhancement ratio at 37% survival (SER37) = 1.29; *p* < 0.001 and *p* = 0.0068 respectively) (Figure 2A,B). ACLY inhibition followed by 2 Gy ionizing radiation (IR) resulted in a significant defect in DNA damage repair, as shown by an increased number of residual 53BP1 foci 24 h post-IR (UT-SCC-5, *p* = 0.0088; UM-SCC-6, *p* = 0.0029) (Figure 2C,D, Supplementary Figure S3C,D). We then determined whether the defect in DNA damage repair could be a consequence of defective homologous recombination using a GFP based reporter system [19]. Pre-incubation with BMS303141 or B02, a Rad51 inhibitor used as positive control of homologous recombination inhibition, resulted in a significant decrease of the GFP positive fraction (*p* = 0.0036), indicating that ACLY inhibition repressed homologous recombination (Figure 2E,F). These data demonstrate that ACLY significantly influences DNA damage repair, which is in line with a previous report that explored the effect of ACLY-mediated histone acetylation on homologous recombination [15]. To investigate whether other functions of ACLY distinct from homologous recombination repair can be involved, we inhibited fatty acid synthetase (FAS), responsible for the production of palmitate from acetyl-CoA and malonyl-CoA, with C75. Contrary to BMS303141, treatment with C75 did not lead to an increase of residual 53BP1 foci in UM-SCC-6 and UT-SCC-5 cells (UM-SCC-6, *p* = 0.933; UT-SCC-5, *p* = 0.195), nor did it cause radiosensitization (*p* = 0.184) (Supplementary Figure S3E–G), indicating that the radiosensitizing effect of ACLY inhibition is independent of fatty acid synthesis.

### 2.3. High ACLY Levels are Associated with Poor Prognosis in HNSCC Patients Receiving Radiotherapy

**Figure 3:**
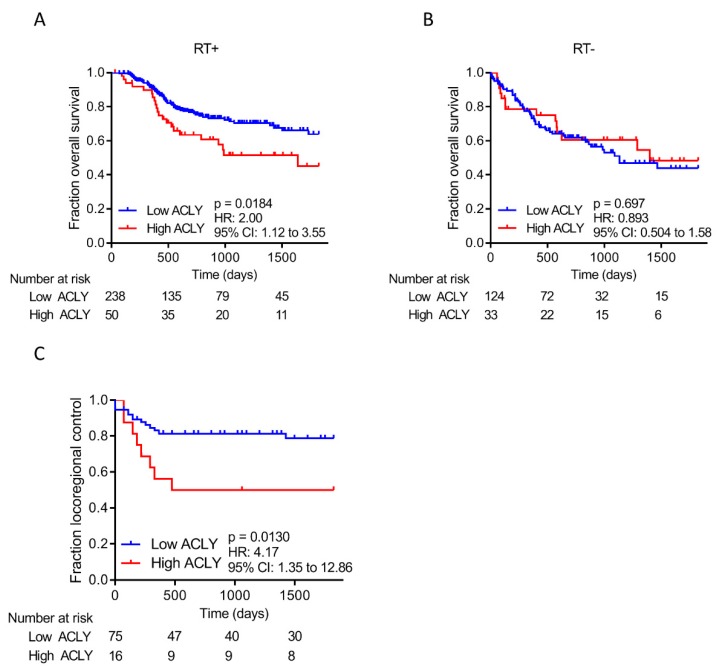
*ACLY* overexpression predicts poor response in RT treated HNSCC patients. (**A**,**B**) Kaplan–Meier analysis of HNSCC patients that have high or low *ACLY* expression based on the optimal cut-off point. Shown are plots for overall survival of HNSCC patients that received radiotherapy (**A**) and patients that did not (**B**). (**C**) Kaplan–Meier analysis of HNSCC patients that have high or low *ACLY* expression based on the previously determined cut-off point. Shown is locoregional control of HNSCC patients that received chemoradiation therapy.

Next, we assessed whether *ACLY* expression levels were associated with HNSCC patients’ responses to RT, using an experimental and validation cohort. For the experimental cohort, we extracted gene expression data of 445 patients from the HNSCC TCGA dataset (Table S2). To objectively stratify patients in two groups (low *ACLY* expression and high *ACLY* expression), we used Cut-off Finder [20] to determine the optimal cut-off point in patients who did or did not receive RT. The optimal cut-off point was determined to be at 81.3% of the population, and was used in all subsequent analyses. Patients that were treated with RT and had high *ACLY* expression, had a significantly worse overall survival than patients with low *ACLY* expression (hazard ratio (HR) = 2.00; 95% confidence interval (CI) 1.12–3.55; *p* = 0.0184) (Figure 3A). In contrast, *ACLY* expression did not correlate with the overall prognosis of patients that did not receive RT (HR = 0.893; 95% CI 0.504-1.58; *p* =0.697) (Figure 3B). Together, these results are in line with our preclinical data suggesting that patients with high *ACLY* expressing tumors have a superior DNA damage repair capacity, leading to radioresistance. Of note, ACLY expression did not affect the recurrence free survival, both in patients who did and who did not receive RT (RT + HR = 1.293; 95% CI 0.684–2.45; *p* = 0.389; RT − HR = 0.804; 95% CI 0.320–2.02; *p* = 0.660) (Supplementary Figure S4A,B). Overall, no major differences were observed regarding TNM status in patients with high *ACLY* expression versus patients with low *ACLY* expression (Table 2).

To validate that patients who received RT have a poorer prognosis when expressing high levels of *ACLY*, we tested an alternate HNSCC patient cohort [21]. In this validation cohort 91 patients were included, all treated with a combination of cisplatin and RT (Supplementary Table S3). Like the TCGA HNSCC cohort, follow-up time was censored at five years. The same cut-off point as for the TCGA cohort was applied (e.g., 81.3%) and survival analysis was performed using Kaplan-Meier analyses. For this cohort, only locoregional control data was available. Again, patients who had a high *ACLY* expression had a significantly worse locoregional control than patients with low ACLY expression, confirming the results of the HNSCC TCGA cohort (HR = 4.17; 95% CI 1.35–12.86; *p* = 0.0130) (Figure 3C). Taken together, this strongly suggests that HNSCC patients who receive RT have an overall worse prognosis when they express high levels of *ACLY*, and that *ACLY* expression is predictive for therapy outcome.

### 2.4. Nuclear ACLY Localisation is Associated with Locoregional Control in HNSCC Patients

**Figure 4:**
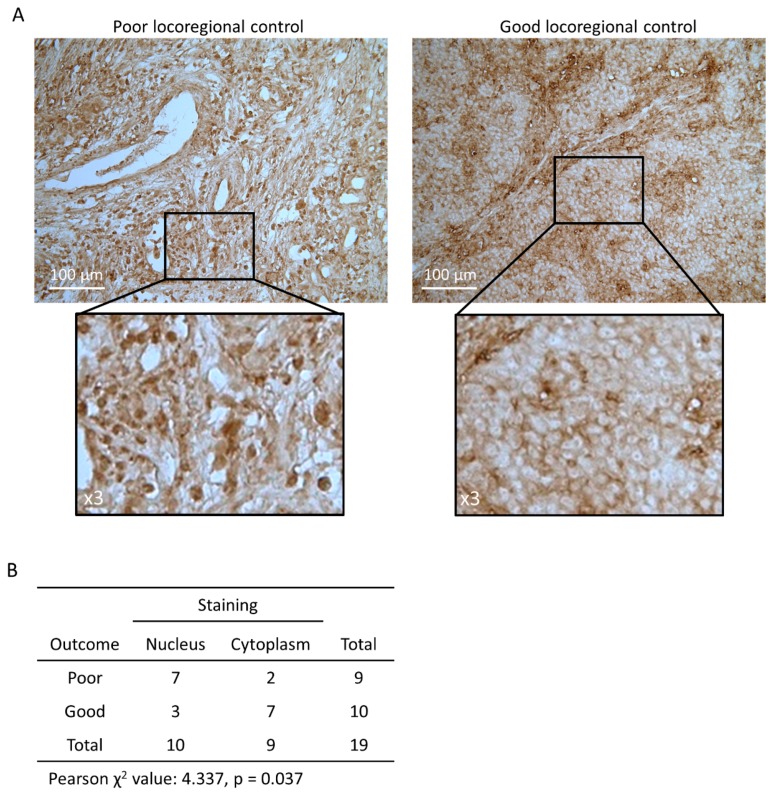
Nuclear ACLY staining is associated with poor outcome in HNSCC patients. (**A**) Immunohistochemistry images showing total ACLY in HNSCC patient biopsy sections. The left pane corresponds to a representative section of a patient with poor locoregional control; the right pane to a patient with good locoregional control. Images are at 20× (top) or 60× (bottom) magnification. (**B**) χ2 test of patients with low vs. high regional control and nuclear or cytoplasmic staining. This is a Figure, Schemes follow the same formatting.

To further investigate the clinical impact of ACLY levels in more detail, we tested the levels of total ACLY protein in biopsies of HNSCC patients. 19 biopsies of HNSCC patients of a previously described cohort [22] were selected for analysis, based on the locoregional control; nine patients with poor locoregional control (locoregional event within 177 ± 36 days), and ten patients with good locoregional control (all minimal recurrence free survival of three years) (Supplementary Table S4). We hypothesized that patients with a poor locoregional control had higher levels of ACLY than patients with a good locoregional control. Overall, ACLY expression seemed ubiquitously expressed in all patient samples, yet demonstrated a marked pattern in terms of cellular localization (Figure 4A). Strikingly, patients with poor locoregional control had significantly higher levels of ACLY in the nucleus (*p* = 0.037). In patients with good locoregional control, ACLY was almost unequivocally expressed in the cytoplasm, but not the nucleus (Figure 4B). This indicates that not only ACLY expression, but also its localization could play a critical role in determining treatment response in HNSCC patients.

## 3. Discussion

In this study, we have shown that a targeted RNA sequencing approach allowed to identify gene transcripts that are associated with radioresistance in HNSCC cell lines. One of the top hits, *ACLY*, correlated with radioresistance, and inhibition of ACLY resulted in impaired homologous recombination, leading to decreased DNA damage repair and radiosensitization. Furthermore, we demonstrated that high *ACLY* expression in patient cohorts corresponds to worse outcome, both for locoregional control as well as overall survival, and that nuclear ACLY is mainly found in HNSCC patients with poor outcome. It has previously been described that *ACLY* is overexpressed in malignant tissues as compared to normal tissues, which could provide for an optimal therapeutic window, yet the cause of overexpression of *ACLY* in HNSCC patients has not been investigated [23]. Experiments performed in visceral adipose tissue suggest that ACLY levels could be elevated as a result of an hypoxic environment, and that *ACLY* could be a possible target gene of the hypoxia-inducible factor 1α (HIF-1α) [24]. In that case, *ACLY* could correlate with the occurrence of hypoxic tumors, and the observed poor treatment response could be also partially attributed to the lack of oxygen required for effective irradiation.

ACLY catalyzes the production of acetyl-CoA and oxaloacetate from citrate and CoA, and thereby plays a crucial role in fatty acid synthesis and acetylation reactions [25]. A number of mechanisms could be proposed for the DNA damage repair deficiency that was observed after ACLY inhibition. Inhibition of general fatty acid synthesis would deprive cells of the macronutrients required for cell proliferation, as well as fatty acids required to repair radiation-associated peroxidised lipids. Inhibition of FAS or FASN expression has been previously shown to sensitize prostate cancer and non-small cell lung cancer cells to irradiation [26,27] However, FAS inhibition did not result in an increase in DNA damage or radiosensitization in HNSCC cell lines. Interestingly, we now show that direct inhibition of ACLY results in impaired DNA repair within hours, specifically homologous recombination. Previously it has been demonstrated that nuclear ACLY promotes the cell’s choice for homologous recombination through elevated levels of acetylated histones at the site of double strand breaks [15]. A shift in DNA damage repair to the virtually error-free homologous recombination pathway, rather than the more error-prone non-homologous end-joining, is likely to result in an improved DNA damage repair efficacy and thereby contributes to radioresistance.

Additionally, we show that high *ACLY* expression is a prognostic factor for worse overall survival in a cohort of HNSCC patients. In line with its apparent role in DNA damage repair, high *ACLY* expression predicted worse outcome only in patients that received RT, but not in patients that did not, thus indicating that *ACLY* is a predictive biomarker for RT success, of which there is a critical need in HNSCC. In a validation cohort where all patients have been treated with chemoradiation (platinum-based chemotherapy and RT), better locoregional control was observed in patients who had low *ACLY* expression levels. This further strengthens the notion that ACLY mediates radioresistance, and thus high levels of *ACLY* result in poor treatment success. Furthermore, while ACLY protein seemed ubiquitously expressed, ACLY localization was strikingly different between patients with good versus poor locoregional control. Localization in the nucleus was significantly associated with poor locoregional control, in line with data from an earlier report that demonstrates that, while acetyl-CoA is able to diffuse through nuclear pores, the activity between nuclear and cytoplasmic acetyl-CoA pools differs [28]. Overall, this supports the hypothesis that nuclear-located ACLY facilitates the production of nuclear acetyl-CoA, which in turn affects the histone acetylation status of the chromatin, and thereby deters the cell’s choice for homologous recombination as the preferred pathway for DNA damage repair [15].

## 4. Materials and Methods

### 4.1. Cell Lines, Reagents, and Irradiation

UT-SCC-5, UT-SCC-8, UT-SCC-9, UT-SCC-11, UT-SCC-15, UT-SCC-19A, UT-SCC-24A, UT-SCC-29, UT-SCC-38, UT-SCC-40, UT-SCC-45, FaDu (Kindly provided by Prof Grenman, University of Turku), UM-SCC-6, UM-SCC-47 (Kindly provided by Dr Carey, University of Pittsburgh), 93-VU-147T (Kindly provided by Dr Dorsman, Amsterdam University Medical Center), and UPCI:SCC-154 (DSMZ) were cultured in DMEM medium (Gibco) supplemented with 4.5 g/L glucose, GlutaMAX, 10% FBS, 100 u/mL penicillin/streptomycin, non-essential amino acids (Gibco), HEPES (Gibco), and sodium pyruvate (Gibco). U2OS DRGFP cells (provided by Prof Tim Humphrey, University of Oxford) were cultured in DMEM supplemented with 10% FCS and 5 mg/mL puromycin and 100 U/mL penicillin/streptomycin. Cells were treated with 5 µM BMS303141 (Sigma-Aldrich, St. Louis, MO, USA) in DMSO or 10 µM C75 (Merck) in DMSO for 6 h prior to irradiation. Single dose irradiation was delivered using a 320 kV XRAD irradiator (RPS Services Limited, Surrey, UK)) at a dose rate of 3.1 Gy/min. An overview of cell line characteristics and tissue origin can be found in Table S1.

### 4.2. Homologous Recombination Assay

U2OS DRGFP cells were plated in a 6 cm dish at a concentration of 2.5×10^5^ cells per well and left to attach overnight. Cells were treated with an ACLY inhibitor (BMS303141, Selleckchem, Munich, Germany) or Rad51 inhibitor (B02, Calbiochem) for 6 h and then transfected with 10 µg pCBASceI plasmid (Addgene #26477) using Lipofectamine 3000 reagent for 24 h. Medium was refreshed after 24 h and cells were harvested by trypsinisation 48 h after transfection. The fraction GFP positive cells was determined by flow cytometry using a FACS Canto II (BD Biosciences, Franklin Lakes, NJ, USA).

### 4.3. Single Molecule Molecular Inversion Probe Sequencing

RNA was isolated from 14 cell lines using TRIzol reagent (ThermoFisher Scientific, Waltham, MA, USA) and reverse transcribed with Superscript II (ThermoFisher Scientific) using random hexamer primers, according to the manufacturer’s instructions. Targeted RNA sequencing using smMIPs has been described in detail before [16,29].

SmMIPs were designed against target regions of interest (UCSC human genome assembly hg19 and splice-variant specific FASTA sequences) based on the MIPgen algorithm as described by Boyle et al., including a random octanucleotide unique molecule identifier [30]. SmMIPs were pooled at 100 µM/smMIP (1078 smMIPs, ~200 transcripts) and phosphorylated using T4 Polynucleotide Kinase (New England Biolabs, Ipswich, MA, USA) in T4 DNA ligase buffer (NEB) for 45 min at 37 °C, followed by 20 min inactivation at 65 °C. Phosphorylated smMIPs were hybridized to 50 ng of cDNA, followed by enzymatic gap-fill by primer extension and ligation in a reaction including Ampligase buffer (Epicentre, Madison, WI, USA), dNTPs, Hemo KlenTaq enzyme (New England Biolabs, NEB, Ipswich, MA, USA) and DNA ligase (Ampligase, Epicentre), incubated for 10 min at 95 °C followed by 18 h at 60 °C. Non-circularized smMIPs and remaining RNA and cDNA were removed by exonuclease treatment with 10 U Exonuclease I and 50 U of Exonuclease III (both NEB) for 45 min at 37  °C, followed by heat inactivation (95  °C, 2 min). The circularized smMIP library was subjected to standard PCR with 2x iProof High-Fidelity DNA Polymerase master Mix (Bio-Rad, Hercules, CA) with a primer set containing a unique barcoded reverse primer for each sample. The pool was then purified using AMPureXP beads (Beckman Coulter Genomics, High Wycombe, UK) according to manufacturers’ instructions. smMIP-PCR libraries were sequenced on the Illumina Nextseq platform (Illumina, San Diego, CA, USA) at the Radboudumc sequencing facility. Reads were mapped against reference transcripts (UCSC human genome assembly hg19 and variant-specific FASTA sequences) using the SeqNext module of JSI SequencePilot version 4.2.2 build 502 (JSI Medical Systems, Ettenheim, Germany). The unique molecule identifier was used to reduce all identical PCR amplification products to one consensus read (unique read). Unique read counts for each smMIP were normalized to the total unique read count within a sample and multiplied by 106 (Fragments per Million, FPM). Individual transcript levels were expressed as mean FPM of all smMIPs targeting that transcript.

### 4.4. Colony Forming Assays

Cells were plated at a density of 150–19,200 cells/well in a 6-well polystyrene culture plate (Corning, Corning, NY, USA). Cells were incubated overnight, treated with BMS303141, C75, or a DMSO control for 6 h and irradiated while still in single-cell phase. Medium was refreshed 24 h after IR and cells were left to form colonies for 8–14 days. Colonies were fixed and stained by crystal violet staining (50% methanol, 20% ethanol, 30% water, 5 mg/mL crystal violet).

### 4.5. Radiosensitivity Analysis and Linear Quadratic Fitting

Statistical analysis was carried out using Prism v8.01 (GraphPad). For colony survival assays the plating efficiency per condition was determined by dividing the total number of counted colonies by the total number of plated cells. Surviving fractions (SF) were calculated by dividing the plating efficiency of treated cells by the plating efficiency of the control cells. Data was fitted according to the linear quadratic model (LQ) using the formula SF = e ^ − (αD + βD^2^). The dose permitting a 37% clonogenic survival (D37) was used as a measure for cellular radiosensitivity and was calculated from the α and β generated by Prism LQ fitting. Sensitiser enhancement ratio (SER) was calculated as SER37 = D (without sensitiser)/D (with sensitiser) for the same biological effect at SF = 37%.

### 4.6. siRNA Interference and Real Time Quantitative PCR (RT-qPCR)

siRNA mediated depletion was performed using the Lipofectamine RNAiMAX system (Invitrogen) according to the manufacturer’s instruction. Cells were transfected for 72 h with scrambled siRNA (SIC002 MISSION esiRNAm Sigma-Aldrich) or *ACLY* targeting pooled siRNA (EHU08192 1MISSION esiRNA, Sigma-Aldrich). RNA isolation and RT-qPCR was performed as previously described [5]. HPRT forward: 5′-TATTGTAATGACCAGTCAACAG-3′; HPRT reverse: 5′-AAGCTTGCTGGTGAAAAGGA-3′; ACLY forward: 5′-TGCTCGATTATGCACTGGAAGT-3′; ACLY reverse: 5′-ATGAACCCCATACTCCTTCCCAG-3′.

### 4.7. Immunofluorescence

5 × 10^4^ cells were plated in 8-well chamber slides (Nunc-Lab-Tek) and incubated overnight. Treatment with BMS303141/C75 was carried out and cells were irradiated with 2 Gy. Cells were fixed in 4% formaldehyde, permeabilized in 1% Triton-X in PBS and blocked in 2% bovine serum albumin in PBS-Tween (0.1%). Cells were subsequently stained with 1:1000 DAPI (Cell Biolabs) and 1:250 53BP1 (Novus NB100-305) primary antibody and 1:500 secondary Fab Cy3 antibody (Jackson ImmunoResearch, Ely, UK). Slides were mounted in fluoromount (Serva Electrophoresis GmbH, Heidelberg, Germany) and imaged on a Leica microscope.

### 4.8. TCGA Data Extraction and Selection Criteria

Xena browser (https://xenabrowser.net/) was utilized to extract data from the HNSC TCGA dataset [31]. From this dataset, radiotherapy status, sample type, time to event (death), event status, recurrence free survival, TNM status, and RNA sequencing expression data of genes of interest were extracted. Samples that were non-primary tumor (e.g., normal tissue or metastatic tissue) were excluded, as well as samples from patients with MX or M1 status of where radiotherapy status was unknown. Data was censored after 5 years follow-up. A total of 445 samples were selected for analysis, of which 288 patients received radiotherapy.

### 4.9. TCGA Data Analysis

Patient and gene expression data was analysed using SPSS v25.0.0.1 (IBM, Armonk, NY, USA). Optimal cut-off points for survival analysis were determined using the Cut-off Finder webtool (http://molpath.charite.de/cutoff/index.jsp). The optimal cut-off point for overall survival was determined as the highest or lowest hazard ratio that was significant (outside the 95% confidence interval) [20]. Based on the optimized cut-off values for expression of the gene of interest, overall survival and recurrence free survival were evaluated using Kaplan–Meier analyses, and tested for statistical significance using the log-rank test. Hazard ratios were calculated between high and low expression groups using a Cox regression model.

### 4.10. Validation Dataset

As a validation cohort, we used clinical data and gene expression profiles of the Pramana HNSCC patient cohort [21]. This cohort contained data of 91 HNSCC patients who were all treated with cisplatin and radiotherapy. An extensive description of the patient cohort can be found in the original publication.

### 4.11. Immunohistochemistry

Of a previously described cohort of HNSCC patients, 19 paraffin embedded biopsies were selected based on locoregional control; 9 biopsies of patients that had a poor locoregional outcome, and 10 biopsies of patients with favourable locoregional control (Table S3) [22]. Sections of 5 µM thickness were cut, deparaffinated and rehydrated. Sections were incubated in citrate buffer (Dako, Santa Clara, CA, USA) at 96 °C for 30 min, cooled, and blocked using 5% normal donkey serum in primary antibody diluent (Bio-rad, Hercules, CA, USA) at room temperature for 30 min. Total ACLY was stained using an ACLY antibody (HPA022434, Sigma-Aldrich) 1:25 at 4 °C overnight, followed by a 0.3% H_2_O_2_ peroxidase block and secondary antibody incubation with 1:200 F (ab) donkey anti rabbit Biotin (Jackson Immuno Research). Sections were then incubated in Vextastain avidin-biotin-complex reagent (Vector), rinsed with water, and incubated with diaminobenzidine (Vector). Afterwards, they were dehydrated and mounted in HistoChoice mounting medium. Images were acquired on a Leica DM 6000 microscope at 20 × magnification. Scoring of nuclear or cytoplasmic staining of total ACLY was performed blindly by two independent researchers after a technician had randomised the images.

### 4.12. Statistics

For in vitro assays, single variable comparisons between two groups were performed on the mean of three biological independent repeats using a two-tailed unpaired t-test with Welch’s correction. For experiments involving multiple variables, an ANOVA was performed, followed by Sidak’s correction for multiple testing. Testing of categorical variables was done using a Pearson’s χ^2^ test. Unless specified otherwise, error bars indicate the standard error of the mean of three biological replicates. Statistical significance is indicated as follows: * *p* < 0.05, ** *p* < 0.01; *** *p* < 0.001.

## 5. Conclusions

As radiotherapy is an important treatment option in HNSCC, it is critical to elucidate the mechanisms that contribute to the development of radioresistance and to devise strategies for targeting this. It is becoming clear that metabolic reprogramming contributes to radioresistance, and affects treatment outcome. Here, we show that nuclear-located ACLY, an enzyme that catalyses the production of acetyl-CoA and oxaloacetate from citrate and CoA, correlates with radioresistance in HNSCC cell lines as well as different patient cohorts treated with (chemo)radiation, and demonstrate that ACLY directly affects DNA damage repair.

## Figures and Tables

**Table 1:** Genes significantly correlating with D37 values of 14 HNSCC cell lines. Spearman’s rho correlation was used to determine the strength of association. Shown are transcripts and their encoded proteins that are associated with radioresistance or -sensitivity.

Gene Name	Encoded Protein	Spearman’s Rho	*p*-Value	High Expression Associated with Radioresistance/Radiosensitivity
*ACACA*	Acetyl-CoA carboxylase alpha	0.596	0.025	Resistance
*ACLY*	ATP citrate lyase	0.670	0.009	Resistance
*ALDOA*	Aldolase, fructose-bisphosphate A	−0.538	0.047	Sensitivity
*BRAF*	B-Raf	0.657	0.011	Resistance
*EGF*	Epidermal growth factor	0.573	0.032	Resistance
*GCLC*	Glutamate cysteine ligase	0.534	0.049	Resistance
*GLDC*	Glycine dehydrogenase	0.650	0.012	Resistance
*GLUL*	Glutamine synthetase	0.666	0.009	Resistance
*GOT1*	Glutamate oxaloacetate transaminase	0.684	0.007	Resistance
*HPV*	E2, E6, E7 (all transcripts)	−0.547	0.043	Sensitivity
*IDH3A*	Isocitrate dehydrogenase 3, mitochondrial, alpha	0.578	0.030	Resistance
*KDR*	Vascular endothelial growth factor receptor 2	0.602	0.023	Resistance
*L2HGDH*	L-2-hydroxyglutarate dehydrogenase	0.604	0.022	Resistance
*MYC*	V-myc avian myelocytomatosis viral oncogene homolog	0.692	0.006	Resistance
*PFKM*	Phosphofructokinase 1	0.640	0.014	Resistance
*RPIA*	Ribose 5-phosphate isomerase A	0.710	0.004	Resistance
*SDHD*	Succinate dehydrogenase complex, subunit D	0.604	0.022	Resistance

**Table 2:** Clinical characteristics of The Cancer Genome Atlas HNSCC cohort. Statistical significance between low versus high *ACLY* expression and RT+ and RT- patients was tested using a Chi-square test. * *p* < 0.05, ** *p* < 0.01; *** *p* < 0.001.

Variable		Low *ACLY*		High *ACLY*		*p*-Value	RT−		RT+		*p*-Value
		*N*	%	*N*	%		*N*	%	*N*	%	
Gender	Female	98	27.1	18	21.7	0.313	55	35.0	61	21.2	0.001 *
	Male	264	72.9	65	78.3		102	65.0	227	78.8	
Clinical T status	T1 + T2	136	37.6	18	21.7	0.141	84	53.5	70	24.3	<0.001 **
	T3 + T4	215	59.4	63	75.9		69	44.0	209	72.6	
	Tx or missing	11	3.0	2	2.4		4	2.5	9	3.1	
Clinical N status	N0	168	46.4	36	43.4	0.975	98	62.4	106	36.8	<0.001 **
	N1-3	178	49.2	45	54.2		54	34.4	169	58.7	
	Nx or missing	16	4.4	2	2.4		5	3.2	13	4.5	
Clinical M status	M0	343	94.8	80	96.4	0.667	152	96.8	271	94.1	<0.001 **
	Mx or missing	19	5.2	3	3.6		5	3.2	17	5.9	
Clinical stage	Stage I + II	82	22.7	14	16.9	0.677	66	42.0	30	10.4	<0.001 **
	Stage III + IV	271	74.8	67	80.7		87	55.4	251	87.2	
	Missing	9	2.5	2	2.4		4	2.5	7	2.4	
Received radiotherapy	Yes	238	65.7	50	60.2	0.344	124	79.0	238	82.6	0.344
	No	124	34.3	33	39.8		33	21.0	50	17.4	

## Supplementary Materials

The following are available online at https://www.mdpi.com/2072-6694/11/12/1971/s1, Figure S1: Characterization of HPV status, radiosensitivity and metabolic pathway expression of 14 HNSCC cell lines, Figure S2: ACLY expression does not differ between HPV+ve and HPV-ve cell lines, Figure S3: CLY, but not FAS, inhibition leads residual DNA damage and radiosensitisation, Figure S4: ACLY is not associated with recurrence free survival outcome, Table S1: Cell line characteristics, Table S2: Clinical characteristics TCGA HNSCC cohort, Table S3: Clinical characteristics of the patients selected from the HNSCC cohort previously described by van der Heijden et al., Table S4: Clinical characteristics of the patients selected from the HNSCC cohort previously described by Liskamp et al.
