# Supplementary material for: ACLY (ATP Citrate Lyase) Mediates Radioresistance in Head and Neck Squamous Cell Carcinomas and is a Novel Predictive Radiotherapy Biomarker
Source: Cancers (Basel). 2019 Dec 7;11(12):1971. doi: 10.3390/cancers11121971 (PMC6966650; doi:10.3390/cancers11121971)
Supplement: Supplementary file 1 [file cancers-11-01971-s001.pdf]

Supplementary Materials: ACLY (ATP Citrate Lyase) Mediates Radioresistance in Head and Neck Squamous Cell Carcinomas and is a Novel Predictive Radiotherapy Biomarker

Table S1. Cell line characteristics.

Cell Line	Age	Gender	TNM Status	Primary Tumour Location	Lesion Type	HPV Status
UT-SCC-5	58	Male	T1N1M0	Tongue	Primary	Negative
UT-SCC-8	42	Male	T2N0M0	Supraglottic larynx	Primary	Negative
UT-SCC-11	58	Male	T1N0M0	Larynx	Primary	Negative
UT-SCC-15	51	Male	T1N0M0	Tongue	Recurrence	Negative
UT-SCC-19A	44	Male	T4N0M0	Glottic larynx	Primary	Negative
UT-SCC-24A	41	Male	T2N0M0	Tongue	Primary	Negative
UT-SCC-29	82	Male	T2N0M0	Glottic larynx	Primary	Negative
UT-SCC-38	66	Male	T2N0M0	Glottic larynx	Primary	Negative
UT-SCC-40	65	Male	T3N0M0	Tongue	Primary	Negative
UT-SCC-45	76	Male	T3N1M0	Floor of mouth	Primary	Positive (HPV33)
UM-SCC-6	37	Male	T2N0M0	Oropharynx	Primary	Negative
UM-SCC-47	53	Male	T3N1M0	Oral cavity	Primary	Positive (HPV16)
93-VU-147T	58	Male	T4N2	Floor of mouth	Primary	Positive (HPV16)
UPCI:SCC154	54	Male	T4N2	Tongue	Primary	Positive (HPV16)

Table S2. Clinical characteristics TCGA HNSCC cohort.

Variable		*N*	%
Gender	Female	116	26.1
	Male	329	73.9
Clinical T status	T1+T2	154	34.6
	T3+T4	278	62.5
	Tx or missing	13	2.9
Clinical N status	N0	204	45.8
	N1-3	223	50.1
	Nx or missing	18	4.0
Clinical M status	M0	423	95.1
	M1	0	0
	Mx or missing	22	4.9
Clinical stage	Stage I + II	96	21.5
	Stage III + IV	338	76.0
	Missing	11	2.5
Radiotherapy	Yes	288	64.7
	No	157	35.3

Table S3. Clinical characteristics of the patients selected from the HNSCC cohort previously described by van der Heijden et al. Categorical values were tested using a Chi-squared test. Differences in age were tested by means of an independent t-test with Welch's correction. Stars indicate statistically significant ($p < 0.05$) differences.

Variable		Low ACLY (*N* = 75)		High ACLY (*N* = 16)		*p*-value
		N	%	*N*	%	
T stage	T1+2	3	4.0	3	24.3	0.081
	T3+4	72	96.0	13	75.7	
N stage	N0	19	25.3	5	31.3	0.889
	N1-3	56	74.7	11	68.7	
Age (years)		59.2 ± 9.7		51.5 ± 10.4		0.013*
Gender	Female	25	33.3	5	31.3	0.872
	Male	50	66.7	11	68.7	

Table S4. Clinical characteristics of the patients selected from the HNSCC cohort previously described by Liskamp et al. A selection of 10 patients with high locoregional control and 9 patients with poor locoregional control was made. Categorical values were tested using a Chi-squared test. Differences in age were tested by means of an independent t-test with Welch's correction. n.a.: not available. * $p < 0.05$.

Variable		Good (*n* = 10)		Poor (*n* = 9)		*p*-value
Age		52.1 ± 10.1		62.1 ± 8.7		0.029 *
		N	%	N	%	
T stage	T1+2	5	50	2	22.2	0.210
	T3+4	5	50	7	77.8	
N stage	N0	2	20	2	22.2	0.906
	N1-3	8	80	7	77.8	
M stage	M0	9	90	9	100	n.a.
	Mx	1	10	0	0	
Clinical stage	I + II	0	0	0	0	n.a.
	III + IV	10	100	9	100	
Gender	Female	1	10	2	22.2	0.466
	Male	9	90	7	77.8	
HPV status (PCR)	Negative	7	70	6	66.7	0.876
	Positive	3	30	3	33.3	

A

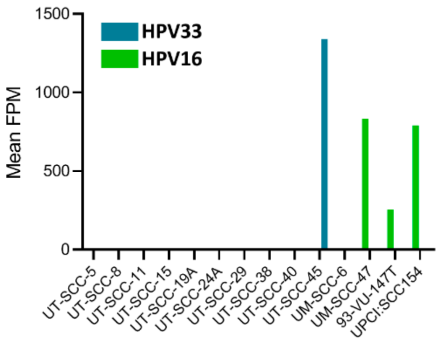

B

Cell line	α	β	Dose for SF_{37}
UT-SCC-5	0.203	0.021	3.56
UT-SCC-11	0.273	0.034	2.72
UT-SCC-8	0.323	0.050	2.28
UT-SCC-15	-0.033	0.043	5.17
UM-SCC-6	0.114	0.105	2.58
UT-SCC-45	0.424	0.057	1.87
UT-SCC-19A	0.190	0.018	3.83
UT-SCC-38	0.443	0.024	2.02
UT-SCC-24A	0.313	0.047	2.36
93-VU-147T	0.668	0.0004	1.49
UT-SCC-29	0.329	0.0331	2.43
UM-SCC-47	0.705	0.0329	1.33
UT-SCC-40	0.670	0.0006	1.48
UPCI:SCC154	0.277	0.083	2.17

C

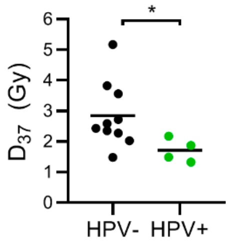

D

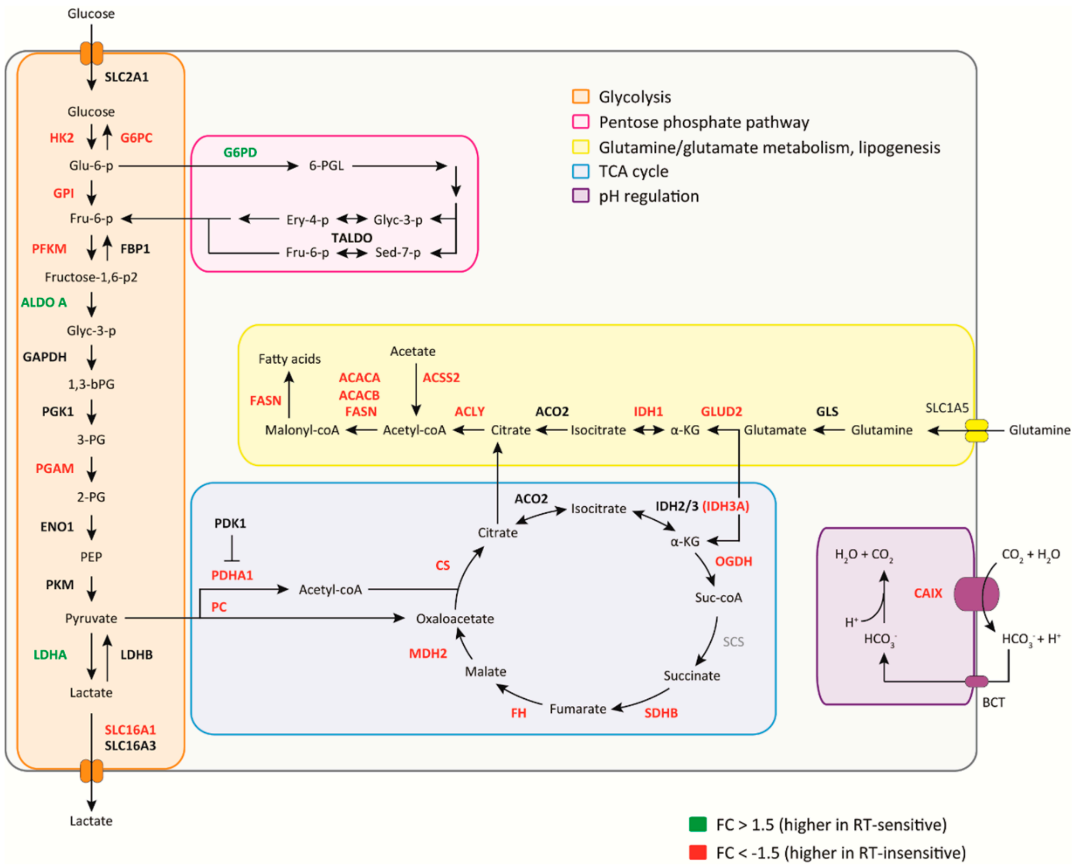

Figure S1. Characterisation of HPV status, radiosensitivity and metabolic pathway expression of 14 HNSCC cell lines. S1A. Expression of high-risk HPV genes E2, E6, and E7 was tested and pooled. 14 HNSCC cell lines were tested for HPV16, HPV18, HPV33, and HPV52. Shown are fragments per million mapped fragments (FPM) for all high-risk HPV types for all cell lines. S1B. Radiosensitivity data of 14 HNSCC cell lines was fitted using the linear quadratic model. The dose permitting 37% survival (D_{37}) was interpolated from this model using the generated *α* and β values. Shown are the *α* and β for each cell line, and the corresponding D_{37}. S1C. D37 data were interpolated from survival curves in 1B using linear quadratic model fitting. D37 data are shown for 10 HPV-ve and 4 HPV+ve

cell lines respectively. Statistical test: two-sided t-test with Welch's correction. S1D. Overview of metabolic pathway expression in four radioresistant vs. four radiosensitive HNSCC cell lines. Shown are genes that were enriched (red) or depleted (green) in radioresistant vs radiosensitive cells by at least a 1.5 fold change in either direction.

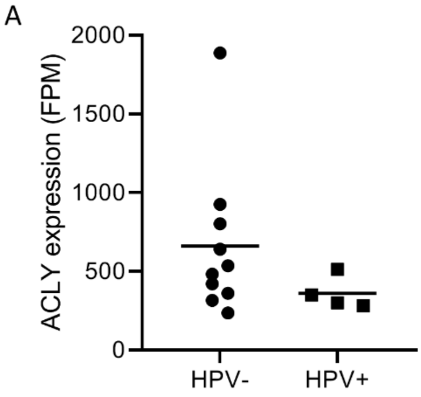

Figure S2. *ACLY* expression does not differ between HPV+ve and HPV-ve cell lines. Gene expression of ACLY was measured in 14 HNSCC cell lines using smMIPs. Comparison of *ACLY* expression between HPV+ve and HPV-ve cell lines was done using an unpaired, two-sided t-test with Welch's correction. FPM: fragments per million.

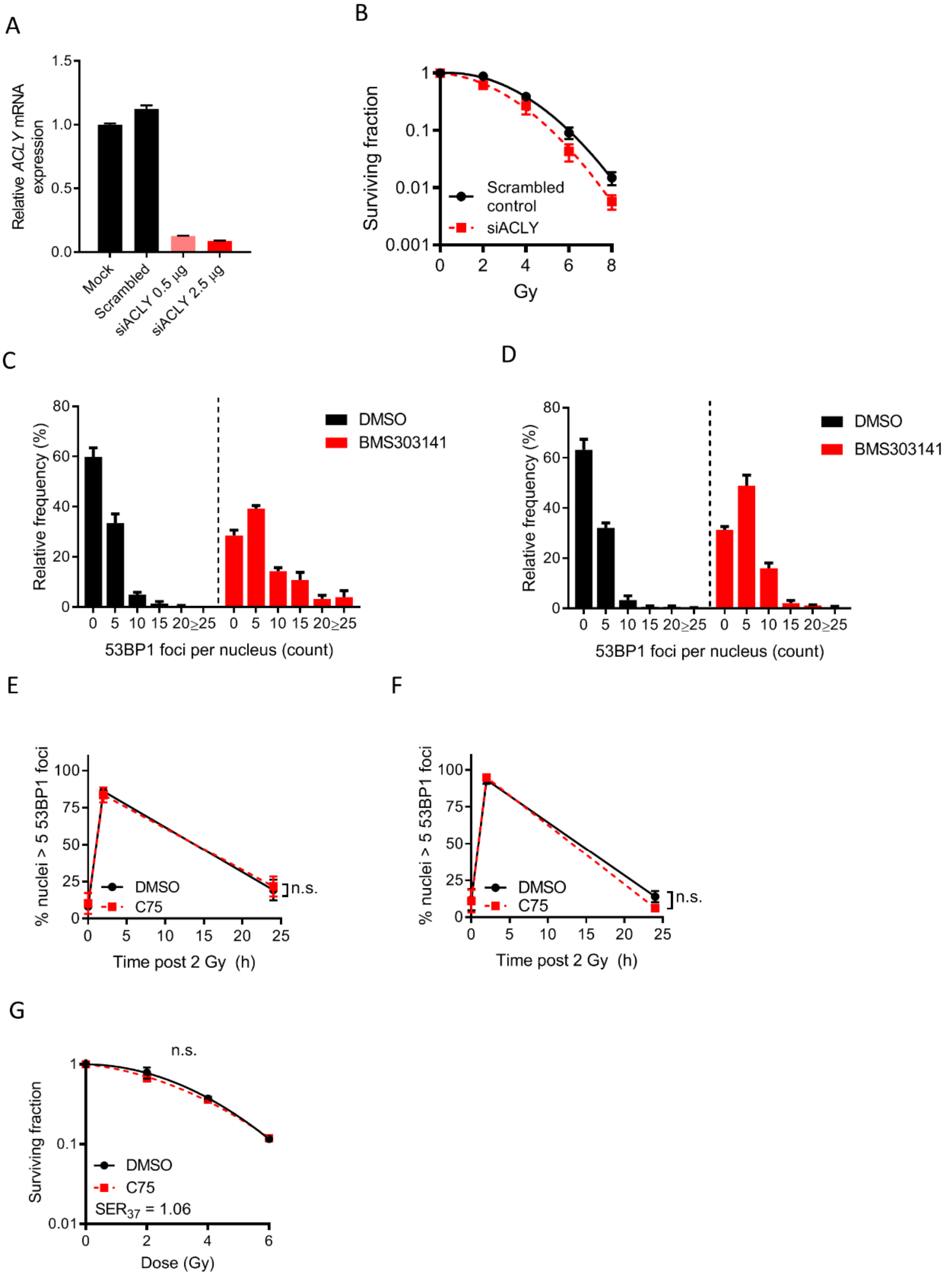

Figure S3. ACLY, but not FAS, inhibition leads residual DNA damage and radiosensitisation. S3A. Depletion of *ACLY* expression in UT-SCC-15 cells was performed using 0.5 or 2.5µg pooled siRNA for 72 hours, followed by confirmation by RT-qPCR. Error bars indicate standard deviation of three technical replicates. S3B. *ACLY* was depleted in UT-SCC-15 cells using pooled *ACLY* siRNA for 72 hours. Subsequently, cells were plated for a colony forming assay and irradiated with the indicated 2, 4, 6, or 8 Gy. Error bars indicate standard deviation of three technical replicates. S3C-D. UM-SCC-6 (A) and UT-SCC-5 (B) cells were treated with 5 µM BMS303141 or DMSO control for 6 hours and irradiated with 2 Gy. 24 hours post-irradiation, quantification of 53BP1 foci was performed. Shown are percentages of cells containing a number of 53BP1 foci per nucleus. Bins indicate the following: 0: 0-4 foci; 5: 5-9 foci etc. SE-F. UM-SCC-6 (C) or UT-SCC-5 cells were treated with 10 µM 10 C75 or DMSO control for 6 hours and were irradiated with 2 Gy. Quantification of cells positive for 53BP1

foci in UM-SCC-6 (C) and UT-SCC-5 (D) cells. Shown are mean percentages of three biological replicates and indicates cells that are positive for 53BP1 (>5 53BP1 foci per nucleus) in a single plane of view. SG. UT-SCC-5 cells were treated with 10 μM C75 or DMSO control for 6 hours and irradiated with 2, 4, or 6 Gy. Survival was determined by colony formation assay. Shown are mean values of two biological repeats with three technical replicates each.

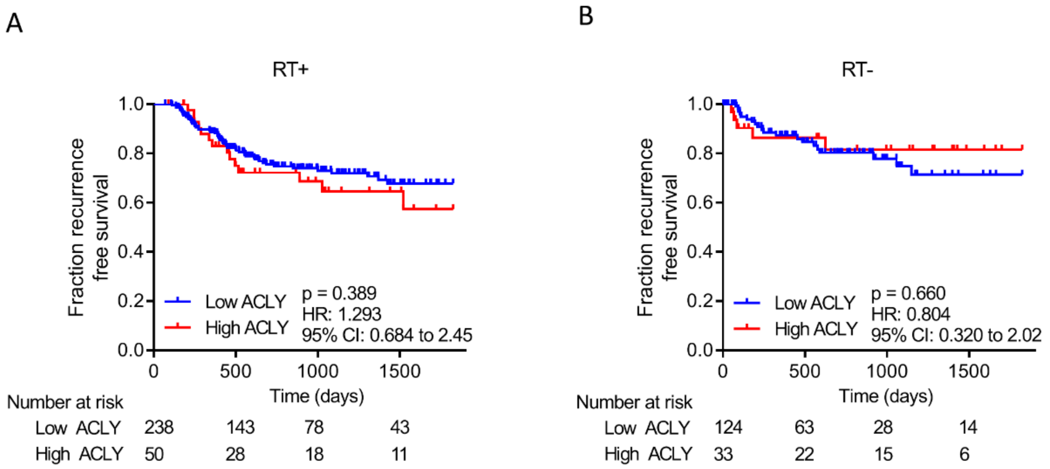

Figure S4. *ACLY* is not associated with recurrence free survival outcome. S4A-B. Kaplan-Meier analysis of a TCGA cohort of HNSCC patients that have high or low ACLY expression based on the optimal cut-off point for overall survival. Shown are plots for recurrence free survival of HNSCC patients that received radiotherapy (**A**) and patients that did not (**B**).

© 2019 by the authors. Licensee MDPI, Basel, Switzerland. This article is an open access article distributed under the terms and conditions of the Creative Commons Attribution (CC BY) license (http://creativecommons.org/licenses/by/4.0/).
